# Short-Term Clinical Outcomes of Robotic and Laparoscopic Cholecystectomy in Indian Patients

**DOI:** 10.7759/cureus.92120

**Published:** 2025-09-12

**Authors:** Sreedhara Setty, Agnigundala Anusha, Ravikiran S J

**Affiliations:** 1 GI, Minimal Access and Bariatric Surgery, Fortis Hospital, Bengaluru, IND; 2 Surgical Gastroenterology and General Surgery, Kauvery Hospital, Bengaluru, IND

**Keywords:** cholecystectomy, learning curve, pain, quality of life, robotic-assisted surgery

## Abstract

Background: This study aims to present the short-term clinical outcomes of robotic-assisted cholecystectomy (RC) compared with laparoscopic cholecystectomy (LC), performed by a skilled laparoscopic surgeon who is just starting the RC learning curve.

Methods: A retrospective medical chart review was conducted for consecutive patients aged ≥18 who underwent non-complex cholecystectomy using either the RC or LC approach between July 2023 and August 2024.

Results: Data from 96 patients (26 in the LC group and 70 cases in the RC group) were collected and analyzed. Baseline characteristics were comparable between the two groups. The mean total operating time was significantly shorter in the RC group compared with the LC group (21.73 ± 12.74 min vs. 32.94 ± 15.51 min; P = 0.0025). The mean length of hospital stay was also shorter in the RC group (P = 0.0368). In the LC group, one bile duct injury was reported. No conversions, postoperative complications, or surgical site infections (SSIs) occurred in either group. The quantity and duration of analgesic use did not differ significantly between the two groups. Pain scores at 24 hours (P < 0.05) and 48 hours (P = 0.0039) were significantly lower in the RC group. Quality of life (QoL) scores were significantly higher in the RC group at seven days (P = 0.0003) and 14 days (P = 0.0108) after surgery.

Conclusion: RC outperformed LC in terms of total operating time, length of stay, pain scores, and QoL scores. Furthermore, a surgeon at the beginning of the RC learning curve can achieve outcomes comparable to those of a surgeon who has already surpassed it.

## Introduction

Gallstones are a common gastrointestinal condition, affecting about 6% of the global population, with a higher prevalence in women [1]. Their rising incidence places a considerable burden on healthcare systems worldwide. Cholecystectomy, whether elective or emergency, is among the most frequently performed surgical procedures for symptomatic or benign gallstones [2]. More than 50,000 cholecystectomies are performed annually in the United Kingdom, while the United States reports nearly 800,000 procedures each year. These figures highlight both the global burden of gallstone disease and the central role of cholecystectomy in its management [1]. Since the mid-1990s, laparoscopic cholecystectomy (LC) has become the preferred approach over open cholecystectomy (OC), due to its clear advantages in reducing hospital stay, morbidity, and mortality, despite its relatively higher intraoperative costs [3,4]. Reported complication rates range from 6.1% to 11.2% for LC, compared with up to 28% for OC [5]. The introduction of robotic-assisted surgery in the mid- to late 2000s, particularly robotic-assisted cholecystectomy (RC), gained popularity because of its advantages, such as wristed instrumentation, improved ergonomics, reduced tremor, and enhanced 3D visualization [6].

RC and LC have shown comparable perioperative outcomes, even when surgeons are still on the learning curve. Similar intraoperative, postoperative, and functional outcomes have also been observed with RC once the learning curve is surpassed [4,7-9]. However, studies consistently report longer operating room times and higher costs for RC. On the other hand, RC has been associated with improved cosmetic satisfaction, body image perception, and pain scores, owing to greater surgical precision, enhanced visualization, and faster recovery [10,11]. In India, the adoption of RC has been relatively slow but has become more common in the past five years. Only a few studies have reported its effectiveness in the Indian context. A case series of 100 patients showed a 3% conversion rate, considered acceptable, along with a steady reduction in operating time as surgeons progressed along the learning curve [12]. Another retrospective study comparing short-term outcomes of RC and LC found similar clinical results, with the RC group reporting less acute postoperative pain [13]. A comparative study using the Parkland Grading Scale demonstrated significant advantages of RC over LC, particularly in higher grades of cholecystitis, intraoperative and postoperative complications, conversion rates, and readmissions [14]. This study aims to present the perioperative and postoperative outcomes of LC and RC for non-complex cholecystitis performed by a skilled laparoscopic surgeon at the start of his robotic surgery practice. The goal is to determine whether a surgeon just beginning the RC learning curve can achieve outcomes comparable to those of a more experienced surgeon.

## Materials and methods

This was a retrospective, non-randomized, comparative study conducted at the Department of Surgical Gastroenterology and General Surgery, Fortis Hospital, Bengaluru, India. The study received approval from the Institutional Ethics Committee and was conducted in accordance with the principles of the latest version of the Helsinki Declaration and applicable good clinical practice guidelines. A medical chart review was performed on consecutive patients aged ≥18 years who underwent non-complex cholecystectomy using either the LC or RC approach between July 2023 and August 2024. Indications for surgery included acute calculus cholecystitis, symptomatic cholelithiasis, and symptomatic gallstone disease. Patients with malignant disease, or those undergoing emergency procedures due to severe complications such as gallbladder perforation or sepsis, were excluded. Records with incomplete or missing follow-up data were also excluded. As part of the institution’s standard practice, all patients were counseled about both RC and LC procedures. The choice of procedure was based on patient preference rather than diagnosis or case complexity. Patients were informed that conversion to an open procedure might be necessary if required. All surgeries were performed according to standard methods recommended by surgical societies. Robotic procedures were carried out using the Da Vinci Xi Surgical System (Intuitive Surgical, Sunnyvale, CA, USA), which features a 3D vision system and endowrist instruments with seven degrees of freedom, allowing precise and flexible movements. Both LC and RC procedures were performed by a single experienced surgeon who had already surpassed the RC learning curve.

Baseline characteristics recorded included age, sex, body mass index (BMI), comorbidities, and history of previous abdominal or pelvic surgery. Perioperative parameters such as total operating time, conversion rates, and intraoperative complications were collected. Postoperative data included length of hospital stay, complications, surgical site infections (SSIs), bile duct injury, analgesic use, pain scores, quality of life (QoL) scores, readmission, and 30-day mortality. Pain scores were measured using the Visual Analog Scale (VAS) [15]. The VAS has a 10-cm line with two endpoints showing the extremes of pain, with "no pain" at the left end (0 cm) and "worst pain" at the right end (10 cm) of the scale. Scores were recorded at 6 hours, 24 hours, 48 hours, 7 days, and 14 days postoperatively. QoL was assessed using the Single-Item Global Quality of Life Scale (SIG-QoL) [16]. This scale has a straight line with separators labeled 10, 20, 30, 40, 50, 60, 70, 80, 90, and 100. The separators are further divided into small lines to show the exact QoL score. The left end of the scale (0) represents the "worst possible" QoL, while the right end of the scale (100) represents the "best possible" QoL.

Quantitative variables were summarized using the arithmetic mean and standard deviation (SD). Categorical variables were summarized as frequencies and percentages. Group comparisons were performed using the Pearson chi-square test or Fisher’s exact test, as appropriate. Differences in means between the RC and LC groups were assessed with a two-sample t-test. A two-sided p-value of <0.05 was considered statistically significant. Statistical analyses were performed using the Stata 16.0 software (StataCorp LLC, College Station, TX, USA).

## Results

Data from 96 patients were included in the study (26 in the LC group and 70 in the RC group). The mean age was 42.30 ± 16.29 years in the RC group and 40.65 ± 11.86 years in the LC group. Females accounted for 70% of cases, and males for 30%, in both groups. There were no significant differences between the groups in terms of age, sex, BMI, comorbidities, or history of abdomino-pelvic surgery. Symptomatic cholelithiasis was a significantly more common indication for cholecystectomy in the LC group compared with the RC group (34.62% vs. 12.86%). No significant differences were observed for the other indications. The descriptive characteristics of the preoperative variables are summarized in Table 1.

**Table 1:** Descriptive characteristics of preoperative variables *Significant value.

Variable	Robotic (N = 70)	Laparoscopic (N = 26)	T-value/χ² value	P-value
Age, mean ± SD, year	42.30 ± 16.29	40.65 ± 11.86	-0.4703	0.6392
Sex, n (%)				
Female	49 (70.00)	18 (69.23)	0.0053	0.942
Male	21 (30.00)	8 (30.77)		
Weight, mean ± SD, kg	65.67 ± 12.37	67.11 ± 9.16	0.4450	0.6580
Height, mean ± SD, cm	157.16 ± 9.26	159.71 ± 9.14	0.9464	0.3483
BMI, mean ± SD, kg/m^2^	27.11 ± 4.63	26.18 ± 3.28	-0.7462	0.4589
Comorbidities, n (%)				
Thyroid disorder	15 (21.43)	3 (11.54)	-	-
Diabetes mellitus	13 (18.57)	4 (15.38)	-	-
Hypertension	10 (14.29)	4 (15.38)	-	-
Previous abdomino-pelvic surgery, n (%)	9 (12.86)	5 (19.23)	0.6183	0.432
Indication for cholecystectomy, n (%)				
Acute calculus cholecystitis	50 (71.43)	15 (57.69)	1.6361	0.201
Symptomatic cholelithiasis	9 (12.86)	9 (34.62)	5.8915	0.015*
Symptomatic gallstone disease	7 (10.00)	2 (7.69)	0.1188	0.730
Acute cholecystitis	3 (4.29)	0	1.1502	0.284
Cholelithiasis	1 (1.43)	0	0.3753	0.540

Table 2 shows the results of the study population's perioperative, postoperative, and functional outcomes. 

**Table 2:** Perioperative, postoperative, and functional outcomes of the study population *Significant value.

Variable	Robotic (N = 70)	Laparoscopic (N = 26)	T-value/χ² value	P-value
Total operative time, mean ± SD, minutes	21.73 ± 12.74	32.94 ± 15.51	3.1174	0.0025*
Length of hospital stay, mean ± SD, days	1.14 ± 0.55	1.62 ± 1.65	2.1177	0.0368*
Conversion, n (%)	0	0	-	-
Intraoperative complications, n (%)				
Bile duct injury	0	1 (3.85)	2.7206	0.099
Postoperative complications before discharge, n (%)	0	0	-	-
Surgical site infection (SSI), n (%)	0	0	-	-
Number of analgesics used in a day, mean ± SD	3.66 ± 1.09	3.65 ± 1.65	-0.0114	0.9909
Pain score, mean ± SD				
6 hours	7.63 ± 1.07	7.96 ± 0.71	1.4552	0.1490
24 hours	4.29 ± 1.40	6.08 ± 1.38	5.5424	0.0000*
48 hours	2.69 ± 1.26	3.58 ± 1.39	2.9611	0.0039*
7 days	0.93 ± 1.11	1.16 ± 0.97	0.9133	0.3634
14 days	0.11 ± 0.36	0.27 ± 0.54	1.5646	0.1212
QoL score at 3 days, mean ± SD				
3 days	57.26 ± 5.63	56.62 ± 5.80	-0.4872	0.6272
7 days	78.37 ± 5.69	73.12 ± 6.67	-3.7415	0.0003*
14 days	89.27 ± 4.50	86.45 ± 3.97	-2.6037	0.0108*
30 days	97.79 ± 1.62	98.32 ± 1.75	1.2033	0.2325
Length of analgesics usage, mean ± SD, days	4.74 ± 0.89	4.92 ± 0.39	0.9886	0.3254
Postoperative complications from discharge up to 30 days, n (%)	0	0	-	-
Re-admission within 30 days, n (%)	2 (2.86)	0	0.7587	0.384
Mortality within 30 days, n (%)	0	0	-	-

In this study, the mean total operative time was significantly shorter in the RC group compared with the LC group (21.73 ± 12.74 min vs. 32.94 ± 15.51 min). The mean length of hospital stay, measured from the day of surgery to discharge, was also shorter in the RC group (1.14 ± 0.55 vs. 1.62 ± 1.65 days, P = 0.0368). In the LC group, one case of bile duct injury was reported. No conversions, postoperative complications, or surgical site infections (SSIs) occurred in either group. Pain management was provided with non-steroidal anti-inflammatory drugs. There was no significant difference between groups in the average number of analgesics used daily (3.66 ± 1.09 vs. 3.65 ± 1.65, P = 0.9909). The duration of analgesic use was slightly shorter in the RC group, but this difference was not statistically significant (4.74 ± 0.89 vs. 4.92 ± 0.38 days, P = 0.3254). No postoperative complications or mortality were observed in either group within 30 days of surgery. Two patients in the RC group required readmission within 30 days; both were admitted for ERCP, and their readmissions were unrelated to RC. Pain scores at 6 hours, 24 hours, 48 hours, 7 days, and 14 days were consistently lower in the RC group compared with the LC group, with statistically significant differences observed at 24 hours (4.29 ± 1.40 vs. 6.08 ± 1.38, P < 0.05) and 48 hours (2.69 ± 1.26 vs. 3.58 ± 1.39, P = 0.0039). Similarly, QoL scores were significantly higher in the RC group at 7 days (78.37 ± 5.69 vs. 73.12 ± 6.67, P = 0.0003) and 14 days (89.27 ± 4.50 vs. 86.45 ± 3.97, P = 0.0108) after surgery.

## Discussion

In recent years, the adoption of RC has steadily increased in India. This retrospective, non-randomized, comparative study reports the perioperative and postoperative outcomes of LC and RC for non-complex cholecystitis. The RC results reflect the initial robotic surgery experience of a skilled laparoscopic surgeon. As there are limited comparative studies on this subject from India, we sought to compare our findings with both the available Indian data and published international literature.

In our study, the RC group outperformed the LC group in terms of total operative time (P = 0.0025) and length of hospital stay (P = 0.0368). By contrast, a prior study from India reported that the RC group had a significantly longer operative time than the LC group (P = 0.0032), although no difference was observed in the length of hospital stay [13]. Another Indian comparative study that included patients with highly complex cholecystitis classified using the Parkland Grading Scale found no significant differences between RC and LC in operative time (1.5 hours vs. 1.25 hours, P = 0.21) or hospital stay (7 days vs. 10 days, P = 0.32) [14]. In our study, one case of bile duct injury was reported in the LC group, while no conversions, postoperative complications, or SSIs occurred in either group. These findings are consistent with a previous Indian study that also reported no conversions or intraoperative complications, along with no significant differences in SSIs and postoperative complications between the two groups [13]. However, our results differ from another Indian study that found the LC group had significantly fewer conversions and intraoperative and postoperative complications than the RC group [14]. A 2024 systematic review and meta-analysis comparing RC and LC [4] found that LC had a significantly shorter operative time (P = 0.0008), with comparable outcomes for bile leak (P = 0.08) and postoperative complications (P = 0.47). Similarly, another systematic review comparing robotic versus laparoscopic multiport cholecystectomy [8] found no differences between the groups in length of hospital stay (P = 0.13), blood loss (P = 0.17), complication rates (P = 0.86), or bile duct injury incidence (P = 0.46). Our findings on hospital stay differ from this review, likely because we calculated length of stay from the date of surgery to discharge, rather than from admission to discharge, to account for delays caused by reimbursement authorization. Contrary to our results, both systematic reviews reported significantly longer operative times for RC compared with LC (mean difference: 10.23 minutes and 25.6 minutes, respectively) [4,8]. In our study, a steady reduction in docking time improved efficiency, which may explain the shorter operative time observed in the RC group. Preventing bile duct injury remains a critical aspect of safe cholecystectomy. While one patient in the LC group in our study experienced bile duct injury, none were observed in the RC group. This finding aligns with several previous studies reporting either a decreased risk or a comparable incidence of bile duct injury with RC [8,17-19].

In our study, the RC group had significantly lower pain scores at 24 hours (P < 0.05) and 48 hours (P = 0.0039) compared with the LC group. The RC group also showed numerically lower scores at 6 hours, 7 days, and 14 days post-surgery. These differences may be attributed to the smaller incisions and the precision of robotic arms. Our findings are consistent with a previous Indian study that reported significantly lower pain scores in the RC group at 24 hours (1.78 ± 0.68 vs. 3.3 ± 1.2, P < 0.001) [13]. Another Indian comparative study also demonstrated lower pain scores in the RC group [14]. Similarly, Lee et al. [11] found significantly lower pain scores in the RC group at 6 hours, 24 hours, 2 days, and 7 days post-surgery. Cho et al. [20] likewise reported that RC patients had significantly lower pain scores than LC patients at two, four, and eight hours after surgery, and that the LC group required significantly higher amounts of analgesics (P = 0.03). Ray and Dhar [13] also observed that opioid analgesics were used at a much higher rate in the LC group (P = 0.0009), whereas the RC group required none. In contrast, our study found no significant differences between groups in either the duration of analgesic use (4.74 ± 0.89 vs. 4.92 ± 0.39 days, P = 0.3254) or the average number of analgesics taken daily (3.66 ± 1.09 vs. 3.65 ± 1.65, P = 0.9909). This may be explained by the fact that, unlike other studies where opioid analgesics were administered, our patients received only non-opioid analgesics.

In our study, QoL scores were significantly higher in the RC group compared with the LC group at seven days (P = 0.0003) and 14 days (P = 0.0108) post-surgery. At the 30-day follow-up, QoL scores in the RC group were slightly lower than those in the LC group, though the difference was not statistically significant. A previous study reported greater body image perception and cosmesis satisfaction in the RC group at all follow-ups but found no statistically significant difference in patient-reported QoL [10]. In our study, two patients from the RC group required readmission within 30 days, though neither readmission was related to the robotic procedure.

Strengths and limitations

To the best of our knowledge, this is the first Indian study to compare the short-term outcomes of RC and LC using data from a surgeon’s initial robotic experience. It is also among the few real-world studies worldwide that directly compare multiport RC with multiport LC. All procedures were performed by a single surgeon, ensuring consistency in surgical workflow. Importantly, the study also reports on functional outcomes, including pain and quality of life. However, certain limitations should be acknowledged, including its retrospective design, case heterogeneity, unequal distribution of patients between groups, and the lack of long-term follow-up data.

## Conclusions

Our study provides a real-world comparison of multiport cholecystectomy performed by a skilled laparoscopic surgeon who was still on the learning curve for robotic surgery. The findings demonstrate that even at the beginning of the RC learning curve, outcomes can be comparable to, or better than, those achieved with LC. In our study, RC outperformed LC in terms of operative time, hospital stay, pain scores, and quality of life, while showing similar results for the amount and duration of analgesic use. These encouraging results highlight the need for larger prospective randomized studies to validate our findings.
